# The Evaluation of the Trueness of Dental Mastercasts Obtained through Different 3D Printing Technologies

**DOI:** 10.3390/jfb15080210

**Published:** 2024-07-29

**Authors:** Lucian Toma Ciocan, Vlad Gabriel Vasilescu, Mihaela Pantea, Silviu Mirel Pițuru, Marina Imre, Alexandra Ripszky Totan, Florin Octavian Froimovici

**Affiliations:** 1 Discipline of Dental Prosthetics Technology, Faculty of Dentistry, “Carol Davila” University of Medicine and Pharmacy, Dionisie Lupu Street, No. 37, District 2, 020021 Bucharest, Romania; lucian.ciocan@umfcd.ro (L.T.C.); florin-octavian.froimovici@drd.umfcd.ro (F.O.F.); 2 Discipline of Prosthodontics, Faculty of Dentistry, “Carol Davila” University of Medicine and Pharmacy, 37 Dionisie Lupu Street, District 2, 020021 Bucharest, Romania; marina.imre@umfcd.ro; 3 Discipline of Organization, Professional Legislation and Dental Office Management, “Carol Davila” University of Medicine and Pharmacy, Dionisie Lupu Street, No. 37, District 2, 020021 Bucharest, Romania; silviu.pituru@umfcd.ro; 4 Department of Biochemistry, Faculty of Dentistry, “Carol Davila” University of Medicine and Pharmacy, Dionisie Lupu Street, No. 37, District 2, 020021 Bucharest, Romania; alexandra.totan@umfcd.ro

**Keywords:** prosthodontics technology, trueness, accuracy, 3D printing, dental mastercasts

## Abstract

In contemporary dentistry, several 3D printing techniques, including a stereolithography apparatus (SLA), digital light processing (DLP), liquid crystal display (LCD), and PolyJet 3D inkjet printing technology (PolyJet), are employed for model production. Despite their widespread use, there remains a paucity of the literature regarding the trueness and precision of these devices in dental applications. Existing studies comparing the accuracy of dental models manufactured by different printing technologies yield disparate conclusions regarding dental prosthesis manufacturing. This study aimed to test two null hypotheses: first, that the trueness of various new-generation 3D printers is equivalent, and second, that the trueness of printing by these printers is sufficient for achieving high-precision mastercasts in dental prosthodontics manufacturing. The research focuses on evaluating the trueness of five contemporary dental 3D printers: Anycubic Mono X 6Ks (Hongkong Anycubic Technology Co., Hongkong, China), Asiga Max (Asiga, Sydney, Australia), Creo C5 (Planmeca Oy, Helsinki, Finland), Form 3B (Formlabs, Boston, MA, USA), and J5 Dentajet (Stratasys Ltd., Eden Prairie, MN, USA). The methodology employed involved the creation of a digital test object using Blender software, adhering meticulously to the dimensions outlined in ISO standard 20896-1. These dimensions were chosen to be both relevant for this study and representative of clinical scenarios. Subsequently, the test object was printed and precise measurements were conducted utilizing a metrology-type Nikon XTH225 ST Reflection target in conjunction with VGStudio MAX analysis software. The results of our investigation revealed clinically negligible deviations in ball dimensions across all printers, with the maximum observed deviations ranging between 1.17% and 2.03% (notably observed in the Creo C5 printer). Transversal distortion exhibited variance based on the linear accuracy of each printer, with Stratasys21 and Formlabs 3B demonstrating superior accuracy among the evaluated printers. Distortions in the analyzed dimensions (specifically, anterior b–c, posterior a–d, and oblique a–c) were found to be uniform. In conclusion, while the first null hypothesis was rejected, indicating variations in trueness among the 3D printers assessed, our findings affirm the suitability of all five analyzed 3D printers for clinical applications. Consequently, these printers can be utilized for the fabrication of high-precision mastercasts in dental prosthodontics manufacturing.

## 1. Introduction

The evolution of digital workflows within dentistry has witnessed a proliferation of clinical applications owing to the advancements in intraoral scanning and additive manufacturing technologies employed within dental laboratories. These scanners serve as conduits for transferring critical information pertaining to a patient’s prosthetic domain into design software, where it is recorded and transformed into digital data, facilitating seamless storage and subsequent utilization throughout the prosthetic fabrication process. This surge in adoption is further underpinned by the strides made in computer-aided design and computer-aided manufacturing (CAD-CAM) technologies, which have revolutionized various facets of dental prosthetic fabrication [1].

Within the realm of digital dentistry, research endeavors often pivot around the evaluation of accuracy, with a keen focus on trueness and precision. Together, these pivotal metrics determine the fidelity of digital computer-aided manufacturing (CAM) objects. Trueness delineates the proximity of the manufactured object—be it milled or printed—to the precise dimensions delineated in the CAD (computer-aided design) model. Put simply, it reflects the degree of convergence between the average measured value and the exact CAD-specified value. Conversely, precision elucidates the reproducibility of achieving consistent results with each fabrication cycle, underscoring the consistency in output. The amalgamation of trueness and precision ultimately delineates the overarching accuracy of the final output [2,3,4].

In recent years, dental models produced via 3D printing technologies have showcased commendable levels of trueness and precision when juxtaposed with their conventional gypsum counterparts [4,5,6,7].

Various 3D printing methodologies, such as a stereolithography apparatus (SLA), digital light processing (DLP), liquid crystal display (LCD), and PolyJet 3D inkjet printing technology, have emerged as stalwarts within the dental domain, leveraging light-induced resin polymerization to craft intricate dental models [8]. Despite the interest, a noticeable lacuna exists within the literature regarding the trueness and precision of dental devices intended for clinical deployment. Existing studies that have endeavored to compare the accuracy of dental models manufactured via diverse printing technologies against their corresponding digital files remain scant in number [9,10,11]. Moreover, these investigations yield disparate findings concerning the clinical significance of deviations across printing platforms.

Recent scholarly discourse has underscored the efficacy of 3D printing in fabricating an array of dental devices—including crowns, surgical guides, and dental models—with clinical outcomes on par with those achieved through traditional milling or production methodologies [12,13,14,15].

The primary objective of this research is to investigate the trueness exhibited by a new generation of dental 3D printers tailored for mastercast fabrication within the realm of dental technology. The assessment encompasses an array of cutting-edge 3D printers, namely the Anycubic Mono X 6Ks (Hongkong Anycubic Technology Co., Hongkong, China), Asiga Max (Asiga, Sydney, Australia), Creo C5 (Planmeca Oy, Helsinki, Finland), Form 3B (Formlabs, Boston, MA, USA), and J5 Dentajet (Stratasys Ltd., Eden Prairie, MN, USA). Through a comprehensive examination of these printing technologies, this study seeks to provide insights into their trueness performance, comparing their results between them but also bringing new conclusions if they are suitable in obtaining 3D-printed models used in dental prostheses’ manufacturing processes.

The primary null hypothesis underpinning this research endeavors to ascertain whether the trueness exhibited by several 3D printers is uniform.

The secondary null hypothesis states that the accuracy of the latest 3D printers enables the creation of high-precision dental mastercasts, thereby meeting the standards necessary for effective dental prosthetic fabrication.

## 2. Materials and Methods

For the purposes of this investigation, a standardized printable object was employed to assess the trueness of the aforementioned state-of-the-art 3D printers that have recently entered the market.

### 2.1. Test Object Creation

**Figure 1:**
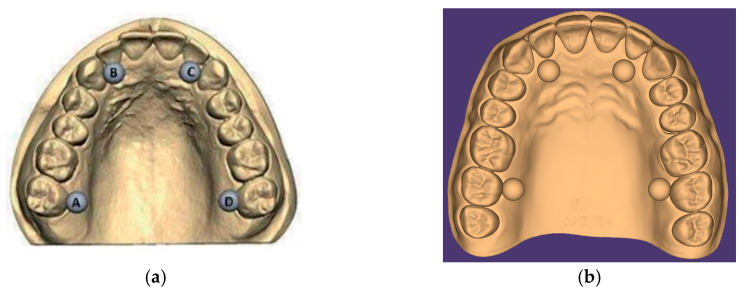
The creation of the digital test object. (**a**) A full-arch test object showing the placement and the sequential designation of four gauge balls [17]. (**b**) The digital test object created in Blender according to ISO standard 20896-1 [17].

The creation of the test object commenced with the digital modeling process facilitated by Blender software (Blender v.3.3 LTS, the Blender Foundation, Amsterdam, The Netherlands) [16], adhering meticulously to the geometric specifications delineated in ISO standard 20896-1 [17] (see Figure 1). This standardized test object for a full dental arch emulates the anatomical structure of either the upper or lower jaw, with complete dentition. Additionally, as per the guidelines outlined in Figure 1, the test object was augmented with the incorporation of four gauge balls composed of a suitable material, affixed immovably at predetermined positions. Notably, the design parameters stipulated by the ISO standard were rigorously adhered to during the digital 3D object’s creation, ensuring precision and fidelity to the specified criteria (refer to Table 1).

The gauge balls, each meticulously crafted to be spherical with a diameter calibrated in accordance with ISO standards, were meticulously designed within Blender software (Blender v.3.3 LTS, the Blender Foundation, Amsterdam, The Netherlands) to possess a size of precisely 6 mm.

In adherence to ISO guidelines, these gauge balls were fashioned from a material chosen for its ability to maintain structural integrity and dimensional stability throughout the testing period. Furthermore, they were seamlessly integrated into the digital model, affixed rigidly from the inception of the design phase to ensure immovability. Any displacement exceeding 0.005 mm would necessitate complete detachment, ensuring stringent control over positional accuracy.

Crucially, the inter-ball distances were varied in accordance with the specifications outlined in the ISO standard, Dentistry-Digital Impression Devices Part 1: Methods for Assessing Accuracy [17]. Detailed specifications regarding the distances between the centers of the gauge balls are delineated in Table 1.

As previously mentioned, a digital test object was crafted using Blender software, aligning with the geometric specifications outlined in the ISO standard. Notably, the dimensions proposed in the second column of the standard were deemed the most pertinent for this study, owing to their relevance and proximity to clinically measurable values. The design process meticulously adhered to the prescribed guidelines, ensuring the precise replication of the digital 3D object. The parameters and values pertinent to the design process are meticulously detailed in Table 2.

The pivotal dimensions under scrutiny for the full dental arch encompass the six distances delineated between the centers of the gauge balls, spanning from point “a” to “b”, “b” to “c”, “c” to “d”, and “d” to “a”, and the two diagonals extending from the center of ball “d” to ball “b” and from the center of ball “a” to ball “c”. This measurement approach facilitates comprehensive insights into both sagittal and transverse dimensions of interest.

Each of these six distances, denoted as D a–b, D b–c, D c–d, D a–d, D a–c, and D b–d, will be measured and documented alongside their respective standard uncertainties, serving as a reference or true values.

In accordance with standardized procedures, the sphericity and calibrated diameter of each gauge ball were considered in determining the precise position of its center. Any deviations from the ideal spherical form were accounted for in the estimation of uncertainties surrounding the dimensions of interest, ensuring robust and precise measurements.

### 2.2. Three-Dimensional Printing of the Test Object 

The test object, a full-arch printed dental model, was fabricated using five distinct new-generation 3D printers (one for each type of printer): Anycubic Mono X 6Ks (Hongkong Anycubic Technology Co., Hongkong, China), Asiga Max (Asiga, Sydney, Australia), Creo C5 (Planmeca Oy, Helsinki, Finland), Form 3B (Formlabs, Boston, MA, USA), and J5 Dentajet (Stratasys Ltd., Eden Prairie, MN, USA).

**Figure 2:**
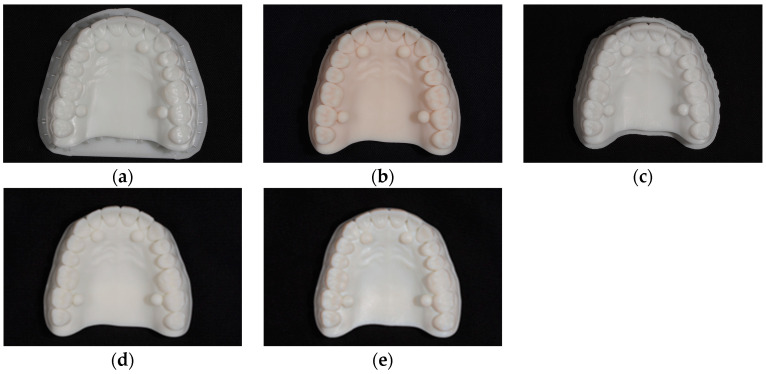
The printed test objects—full arch: (**a**) The test object obtained by Anycubic Mono X 6Ks. (**b**) The test object obtained by Asiga Max. (**c**) The test object obtained by Creo C5. (**d**) The test object obtained by Form 3B. (**e**) The test object obtained by J5 DentaJet.

Our primary focus was directed towards evaluating the print precision relative to the digital file, with the test object serving as a comprehensive representation of a full dental arch. This examination aimed to discern the fidelity of the printed models in faithfully reproducing the intricate details delineated in the digital design file (refer to Figure 2).

To better understand the conditions in which every printer was used, we will describe the parameters of every device.

The Anycubic Mono X 6Ks, developed by Hongkong Anycubic Technology Co., China, is an advanced monochrome LCD-based mSLA printer operating at a wavelength of 405 nm. It features an effective pixel size of 34.4 microns and a resolution of 6K (5760 × 3600) within a 9.1-inch screen. Notably, the layer height is adjustable, commencing from a mere 10 microns. This printer operates a completely open system, accommodating both proprietary software, such as Anycubic Photon Workshop 3.0, as well as third-party software for slicing and printing.

In our experiments, we utilized White-Colored UV Resin from Hongkong Anycubic Technology Co., China, with a layer height set at 50 microns, sliced using Chitubox Basic software from Shenzhen CBD Technology Co., China. The manufacturer offers dedicated washing and curing units to streamline post-processing procedures. Models were subjected to a 10 min immersion in 99% isopropyl alcohol for thorough cleaning, followed by a curing process lasting 10 min in the Form Cure unit from Formlabs, USA, without heating [18,19].

The Asiga Max, developed by Asiga in Australia, operates on digital light processing (DLP) technology, utilizing a dual wavelength of 385/405 nm with a 62-micron pixel size. The printer accommodates both proprietary and third-party resins, facilitated through the Asiga Composer software. Additionally, it features a curing chamber, the Asiga Flash, which exposes printed models to 405 nm light for curing, albeit without heating.

Our experimentation with the Asiga Max involved printing a model utilizing DentaMODEL resin from Asiga Australia. This model was printed at a layer height of 25 microns. Post-print processing adhered to the manufacturer’s guidelines, entailing a cleaning regimen involving immersion in 99% isopropyl alcohol for ten minutes, followed by a subsequent ten-minute immersion in fresh IPA. Subsequently, the model was left to air-dry for a minimum of 10 min to ensure the complete evaporation of the residual solvent before undergoing a curing process in the Asiga Flash unit for 20 min [20].

The Creo C5, engineered by Planmeca Oy in Finland, adopts a monochrome LCD approach, operating at a wavelength of 385 nm with an XY resolution of 50 microns. This printer facilitates the utilization of partnered third-party materials within a closed system, either via branded resins or proprietary cartridges, managed through the Planmeca Creo C5 Studio software. The layer height selection is contingent upon the resin employed and can be finely adjusted within the software interface, offering a range between 25 and 100 microns [21].

In our experimentation, we utilized FotoDent model 2385 nm resin from Dreve Dentamid GmbH in Germany, with a selected layer height of 50 microns. Notably, Planmeca Oy does not incorporate dedicated washing and curing stations; however, the resin manufacturer provides comprehensive guidelines for washing and curing protocols utilizing both a proprietary and third-party apparatus. For our models, washing was executed using the Form Wash unit from Formlabs, USA, employing two cycles of six minutes each in clean 99% isopropyl alcohol. Subsequently, the models underwent a curing process using the Form Cure unit from Formlabs, USA, for a duration of 12 min [21].

The Form 3B, developed by Formlabs in the USA, operates on stereolithography (SLA) technology, utilizing a 405 nm laser with an 85-micron laser spot size and 25-micron XY resolution. This printer is designed to accommodate proprietary materials, dispensed from cartridges, with the software, PreForm by Formlabs, restricting the use of third-party materials. The layer height is adjustable based on the chosen resin type, offering options for precise or expedited printing within the software interface.

For our experiments, we employed Model Resin from Formlabs, utilizing a layer height of 50 microns. Formlabs provides automated washing and curing solutions tailored to each of their proprietary resins, including heating for the curing station. Models printed on the Form 3B underwent a cleaning process in 99% isopropyl alcohol for 10 min using the Form Wash unit. Subsequently, the models were air-dried before undergoing a curing process for 5 min at 60 °C in the Form Cure unit, adhering to the manufacturer’s recommendations [19].

The J5 DentaJet, manufactured by Stratasys in the USA, represents a resin PolyJet 3D printer renowned for its precision, offering a layer thickness as fine as 18 microns and printing resolutions of either 18.75 or 20.625 microns. This printer operates within a closed system, utilizing resin cartridges in conjunction with proprietary software, GrabCAD Print from Stratasys, USA. Noteworthy is its capability to print multi-material parts, leveraging Stratasys’ proprietary range of materials.

In our experimentation, the model was printed using TrueDent White resin from Stratasys, USA, utilizing the 20.625-micron printing profile. Post-printing, the model underwent a cleaning process adhering to the manufacturer’s protocol. This process entailed thorough cleaning with a waterjet, followed by immersion in a 2% caustic soda solution for a duration of 2 h. Subsequently, the model was rinsed and cured utilizing TrueDent Cure from Stratasys, USA, within glycerol at 80 °C for two hours, with each side of the device receiving one hour of curing. The parts were then rinsed in tap water before undergoing a 30 min immersion in 99% isopropyl alcohol, after which they were left to air-dry [22].

It is worth noting that some manufacturers assert that post-processing procedures do not impact the trueness of printed parts, emphasizing their role in reducing residual monomers and optimizing biological and mechanical properties [23].

Due to the known shrinkage and deformation of the resins used with 3D printing, the time of the analysis and digitalization of the printed models was within 48 h after the post-processing protocol was finished. Subsequently, the digital files were analyzed using VGStudio MAX 2023.1. The alignment and superposition of the models were performed with the help of the CT Pro 3D—Nikon software—in two phases: an initial alignment of all the obtained images after scanning and combining them in order to obtain the 3D model. 

In order to more easily understand the specifications and characteristics of each of these 3D printers described previously, we have summarized them in Table 3.

### 2.3. Analysis 

**Figure 3:**
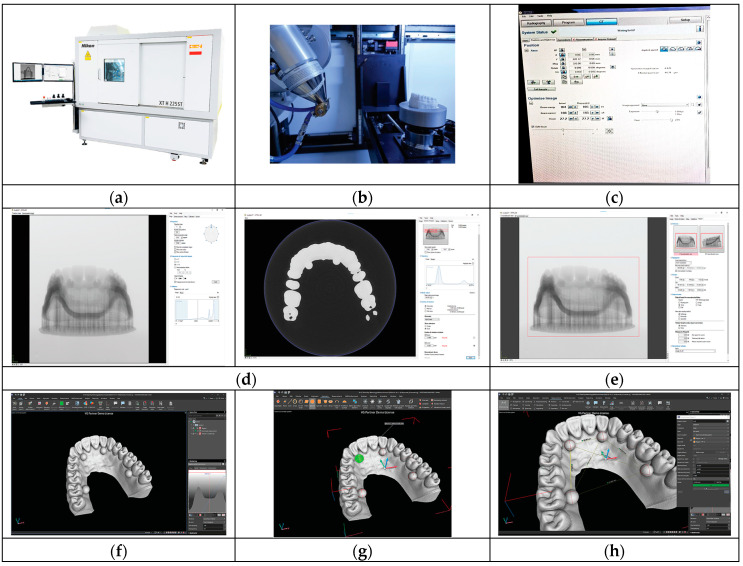
(**a**) Nikon XTH225 ST Reflection target. (**b**) Different samples positioned on the tray inside the equipment. (**c**) Parametrization. (**d**) Model reconstruction. (**e**) Area of interest. (**f**) Imported model. (**g**) Geometry Elements module. (**h**) Measurement module.

Working mode:

Equipment used: Nikon XTH225 ST Reflection target (Figure 3) (Table 4). Analysis software: VGStudio MAX 2023.1. First step: Positioning the sample on the tray inside the equipment. Second step: Parametrization—the configuration and definition of system parameters. Step three: After completing the scanning process, the model reconstruction is carried out using the CT Pro 3D program—by Nikon. In this program, alignment of all the images obtained during scanning is performed, followed by their combination to obtain the 3D model. Step four: After alignment, the selection of the area of interest to be exported is performed. Step five: The model is imported into the VGStudio MAX 2023.1 software, where brightness and contrast are adjusted to achieve the best image quality, followed by taking measurements. Step six: To measure the diameter of the spheres, the Geometry Elements module is used. By selecting the option for sphere identification and placing points on the surface of each sphere, the program can identify them and provide information regarding their diameters. Step seven: To measure the distances between spheres, we use the Measurement module, which allows us to select the type of measurements we want to perform and the reference elements we want to use (in our case—the spheres). Nominal values and tolerances can be entered, and the program can indicate whether our dimension is within tolerance or not.

## 3. Results

The raw data measurement results for each of the printed test objects (printed models), reported to the ISO standard 20896-1 values and nominal values of the digital test object, are listed in Table 4.

The raw data measurement results have been transformed for the calculation of the trueness and trueness deviation (Table 5).

### 3.1. Three-Dimensional Dimension Trueness

**Figure 4:**
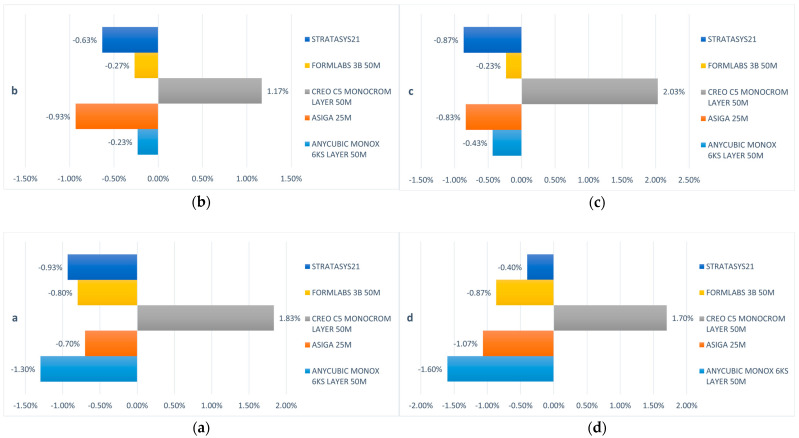
Ball size printing accuracy deviations for each ball (**a**–**d**).

The first null hypothesis was rejected, indicating that the trueness of the last-generation 3D printers investigated in this study was not uniform. The 3D dimension printing trueness is interpreted by ball size of each printed test object (printed models) reported to nominal values of the digital test object. The trueness deviations in ball printing can be seen in Figure 4.

**Figure 5:**
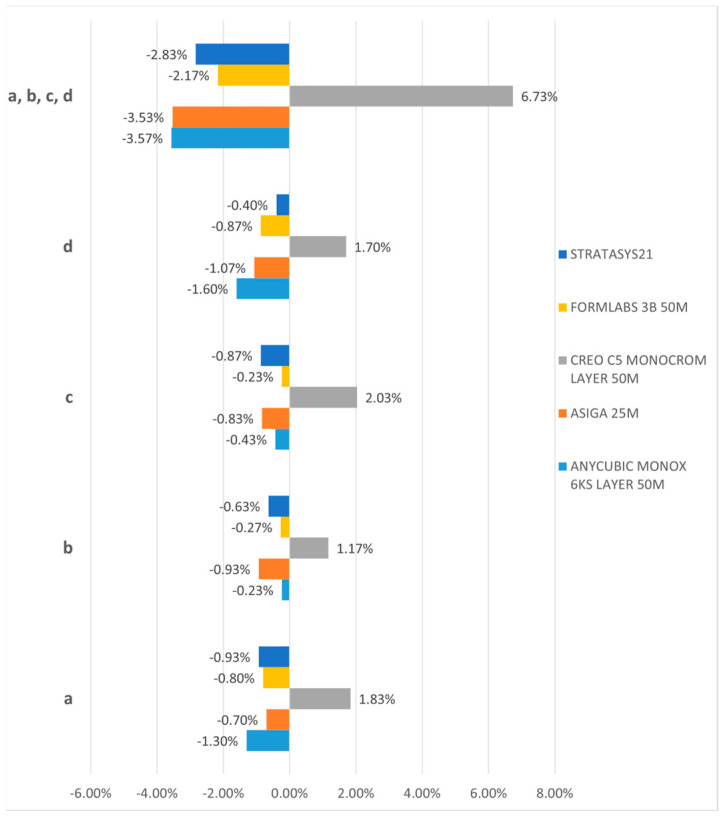
Trueness of the printed models.

All printed test objects (printed models) exhibit deviations in accuracy, particularly noticeable at the level of the printed balls. Notably, the balls printed by the Creo C5 (Planmeca Oy, Helsinki, Finland) exhibit a positive size deviation, whereas those printed with the other printers demonstrate a negative deviation. Specifically, the Anycubic Mono X 6Ks (Hongkong Anycubic Technology Co.), Asiga Max (Asiga), Form 3B (Formlabs), and J5 Dentajet (Stratasys Ltd.) all showcase this negative deviation. Interestingly, no significant disparities were observed concerning the distance of the balls within the printed model, whether anterior or posterior, across different segments of the arch for any of the investigated samples. The comprehensive deviation analysis of the ball sizes is illustrated in Figure 5.

Among the investigated samples, the minimum deviations were recorded for the printed test object (printed models) obtained with the Form 3B (ranging from 0.23% to 0.87%) and the J5 Dentajet (ranging from 0.4% to 0.93%). Subsequently, the deviations for the Asiga Max ranged from 0.7% to 1.07%, while those for the Anycubic Mono X 6Ks ranged from 0.23% to 1.6%. Conversely, the Creo C5 exhibited the maximum deviations, ranging from 1.17% to 2.03% concerning the 6 mm diameter of the balls. It is worth noting that despite these variations, the maximum lack of trueness observed in the printed samples is 120 microns, a value that at first glance may seem significant in terms of the clinical use of these printers, but it can be easily corrected or compensated for by a careful calibration of the protocol and working steps. These values might seem significant, but they do not influence the clinical use of printed models, nor do they restrict the use of printers in various clinical applications.

### 3.2. Linear Trueness and Transversal Distortion

**Figure 6:**
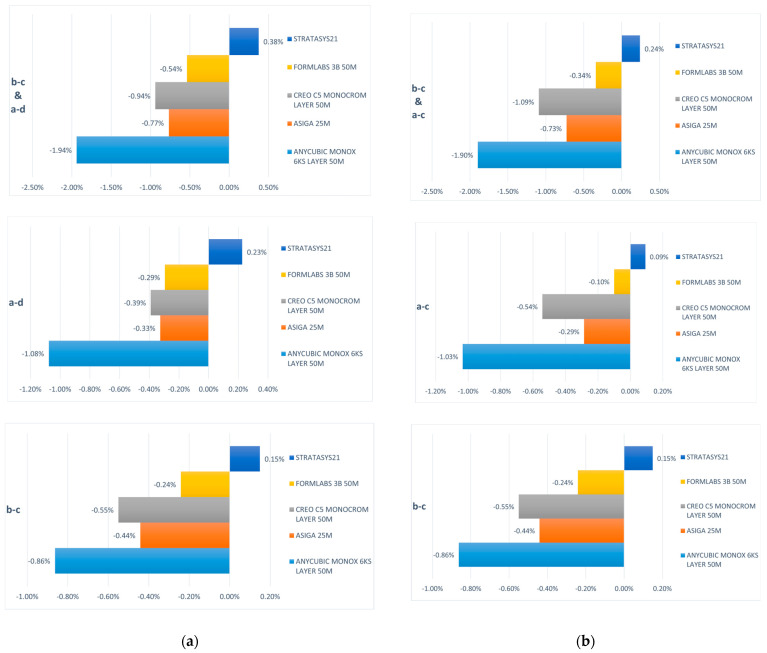
Linear trueness deviations of the printed models (**a**) b–c and a–d, (**b**) b–c and a–c.

To assess linear trueness and potential transversal distortions of the 3D-printed models, an analysis of the distances from the centers of the balls was conducted. The deviations in linear trueness of the printed models are visually represented in Figure 6, where the values correspond to the 3D trueness deviations.

Across all dimensions examined, the J5 Dentajet and Form 3B printers consistently demonstrated the highest levels of accuracy, followed by the Creo C5, and subsequently the Asiga Max and Anycubic Mono X 6Ks. Notably, upon a comparative analysis of anterior, posterior, and antero-posterior dimensions (b–c, a–d, and a–c distances), no significant differences that may restrict the use of printers in various clinical applications were observed, indicating minimal to no distortions present in the printed models. Moreover, the linear deviation values exhibited proportionality for the same printer across all directions, whether sagittal, transversal, or oblique (refer to Figure 6).

## 4. Discussion

Indeed, the accuracy of additive manufacturing in dentistry is influenced by a trifecta of factors: the chosen technology, printer, and material. Each element plays a crucial role in determining the final accuracy of the manufactured device. Throughout the manufacturing workflow, from virtual design to post-processing, the accumulation of distortion at each step significantly impacts accuracy [24,25].

Operator decisions during manufacturing processes, such as virtual design control, parameter selection, slicing techniques, and post-processing application, wield substantial influence over the device’s features and accuracy. For instance, inadequate control over virtual device design, improper thickness selection, or the utilization of suboptimal printing parameters can all lead to compromised accuracy. Similarly, the meticulous application of post-processing techniques is paramount in ensuring that the final device meets the desired specifications.

In essence, the interplay of these factors underscores the importance of operator expertise and meticulousness throughout the additive manufacturing process, as they ultimately dictate the accuracy and functionality of the dental device produced.

The ISO standards provide a clear definition of accuracy, delineating it through two distinct terms: precision and trueness [2]. Precision refers to the degree of repeatability exhibited by a 3D printer when producing an object under identical conditions, whereas trueness pertains to the printer’s ability to construct a CAD object with dimensions closely mirroring its digital counterparts. When assessing accuracy, it is imperative to consider measurements along the *x*-, *y*-, and *z*-axes, where the z-axis represents the vertical plane, and the *x*- and *y*-axes represent the horizontal planes. Furthermore, both precision and trueness of a printer hinge on its resolution, particularly the smallest feature size it can accurately reproduce in both horizontal and vertical dimensions.

Various classifications exist within additive manufacturing, encompassing material extrusion, vat polymerization, material jetting, powder bed fusion, sheet lamination, and direct energy deposition [26]. However, in the field of dentistry, specific additive manufacturing categories are predominantly employed for fabricating polymeric, metal, and ceramic dental devices [27,28,29,30,31]. This selective utilization ensures adherence to stringent standards and enables the production of high-quality, clinically viable dental prosthetics.

The accuracy of 3D-printed models in dentistry hinges on a multitude of factors throughout all processes, where errors may manifest. These factors include parameters employed during manufacturing and post-processing procedures, as well as the acquisition and processing of data relating to hard and soft oral tissues.

A study titled “A comparison of trueness and precision of 12 3D printers used in dentistry” [32] offers valuable insights into the comparative accuracy evaluation of 3D-printed dental models utilizing a diverse array of printers. These printers include the Ackuretta Sol, Anycubic Photon and Photon S, Asiga Max UV, Elegoo Mars, Envisiontec Vida HD, Envisiontec One, Envisiontec D4K Pro, Formlabs Form 2 and Form 3, Nextdent 5100, and Planmeca Creo. Notably, this selection caters to a wide range of budgets, ensuring accessibility across various practice settings. Through this comparative analysis, researchers endeavor to shed light on the nuances of accuracy across different 3D printing platforms, aiding clinicians in making informed decisions regarding printer selection for their specific needs.

While discernible differences in mean error between the printers were identified, the overall performance of these printers is regarded as exceptional. Notably, the Envision One, Envision D4K, Ackuretta Sol, and Asiga Max UV printers emerged as top performers, demonstrating overall trueness under 35 μm. This study unequivocally affirms that all 3D printers examined are capable of producing reliable and reproducible models [32].

When categorized into homogeneous subsets, the more economical 3D printers within the group, specifically the Anycubic printers and the Elegoo Mars, exhibit no statistically significant differences compared to the higher-priced Envisiontec and Asiga Max UV printers, or even the mid-priced Ackuretta and Formlabs printers, in terms of X and Z dimensions. However, the Envisiontec One and D4K Pro, Ackuretta Sol, and Asiga Max UV printers showcased statistical superiority in precision, particularly demonstrating consistently accurate Y dimensions. It is noteworthy that despite employing diverse printing technologies, no specific type of printer technology emerges as inherently more accurate than others [32].

Furthermore, significant findings are also presented in studies concerning the comparative assessment of accuracy in the production of dental models obtained through 3D printing technologies. Additionally, studies delve into the precision of various 3D printing workflows for dental models, comparing industrial printers utilized in dental laboratories with dental office printers [1,32]. These comprehensive analyses contribute to a nuanced understanding of the capabilities and limitations of different 3D printing technologies within the realm of dentistry.

The study titled “Accuracy Comparison among 3D-Printing Technologies to Produce Dental Models” [1] delves into the assessment of different 3D printing technologies, including SLA, DLP, LCD poly- and monochromatic, and Polyjet. Fifteen models were manufactured utilizing five distinct 3D printers, and their accuracy was evaluated through physical measurements of corresponding lengths for trueness and reproducibility. Means and standard deviations were derived for the five computer-aided manufacturing (CAM) methods and subjected to a comparative analysis.

The study’s findings indicate that while 3D printing methods may exhibit minor but statistically significant discrepancies when compared to the original digital files, these variations may not bear clinical relevance. Moreover, no significant disparities were observed among median measurements of each printing method within the 3D printer analysis, suggesting that, for dental casts, all studied devices performed comparably. This underscores the overall reliability and consistency of the various 3D printing technologies examined in the context of dental model production.

The article titled “Accuracy of five different 3D printing workflows for dental models comparing industrial and dental desktop printers” [13] aimed to assess the accuracy, specifically in terms of trueness and precision, of printed models using five distinct industrial and dental desktop 3D printers. Among the industrial printers, the 3D Systems Project MJP2500 (3DS) and Objet30 OrthoDesk (Obj) were evaluated, while the dental desktop printers included the NextDent 5100 (ND), Formlabs Form 2 (FL), and Rapidshape D30 (RS). A total of 225 printed models underwent an analysis.

The printed models were digitized and compared with a reference cast model using Control X software (Geomagic). Descriptive statistics were employed, and one-way ANOVA with a post hoc Tukey test was conducted (α = 0.05). The results of the one-way ANOVA for the trueness and precision of the printed models indicated superior performance by the 3DS, followed by ND, Obj, FL, and RS (*p* < 0.01). In the scanbody zone, the 3DS group yielded the best results, followed by Obj, ND, FL, and RS (*p* < 0.01).

Moreover, when comparing technologies, the Multijet technology employed in industrial printers demonstrated superior results compared to the DLP and SLA technologies utilized in dental desktop printers (*p* > 0.01). These findings provide valuable insights into the comparative performance of different 3D printing workflows, shedding light on the capabilities and limitations of various printing technologies in the realm of dental model fabrication. Statistically significant differences were observed in terms of the accuracy of printed models, with industrial printers exhibiting superior results compared to dental desktop 3D printers. From a clinical perspective, industrial 3D printers utilized in dental laboratories demonstrated higher accuracy than in-office dental desktop 3D printers. This disparity in accuracy should be taken into consideration, particularly when utmost precision is required for final prosthetic restorations.

A systematic review titled “Accuracy of 3-Dimensionally Printed Full-Arch Dental Models” [7] lends further support to the utilization of 3D-printed dental models, particularly in orthodontic study models. Regardless of the 3D printing technology employed, certain printers showcased minimal errors, thereby warranting their recommendation for dental applications demanding high-accuracy models.

The most prevalent 3D printing technology examined across the included studies was SLA, with findings indicating that both SLA and DLP achieved the highest accuracy for full-arch models. Notably, among SLA printers, the Form 2 by Formlabs garnered considerable attention and consistently produced clinically acceptable models. These findings underscore the efficacy of SLA and DLP technologies in achieving accurate and clinically viable dental models, with the Form 2 standing out as a reliable option for dental applications.

Although a broader spectrum of mean errors was observed among SLA printed models, the Form 2 SLA desktop printer [32,33,34,35] consistently outperformed material jetting printers and proved to be more cost-effective [32,36].

Furthermore, this study concluded that the accuracy of 3D-printed models was influenced by the printing technique irrespective of the base design. However, due to limited studies assessing the accuracy of BJ [37] and CLIP technologies [38], further investigation of these techniques is warranted to validate the viability of these printers.

Additionally, factors such as layer thickness, base design, post-processing, and storage can equally impact the accuracy of the resultant 3D-printed models [39,40].

It is noteworthy that some studies did not provide details regarding sample size calculation, resin materials, and/or post-curing protocols, rendering them susceptible to a high risk of bias and applicability concerns regarding sample selection. Consequently, no conclusions were drawn based on these parameters, except for studies that adhered to the manufacturer’s recommendations.

The absence of standardized reporting across different published studies also represents a limitation, potentially leading to a high risk of subjectivity in terms of testing and sample selection. This underscores the need for greater consistency and transparency in reporting methodologies across research in this field.

Consequently, due to the evident heterogeneity among the included studies, characterized by varying techniques, manufacturing parameters, materials, and assessment protocols, conducting a meta-analysis was not feasible. It is imperative to acknowledge the limitations present in the literature, which necessitate addressing in future studies.

Key areas for further investigation include exploring different layer thicknesses for FFF, MJ, BJ, and CLIP printing technologies, assessing the impact of time and storage conditions on the accuracy of different 3D-printed models, and evaluating clinical patient outcomes. Moreover, the development of a standardized accuracy assessment protocol for 3D printing of dental models is essential to facilitate performance comparison across studies. Future research endeavors should also incorporate a standardized reporting protocol detailing all printing parameters, materials used, post-processing protocols, and the timing of assessments.

Consequently, the findings gleaned from the studies cited underscore the significance of employing 3D-printed dental models across various printing technologies. Certain printers have demonstrated reduced errors and may thus be recommended for dental applications necessitating high-precision models. Nevertheless, it is crucial to recognize that factors such as layer thickness, base design, post-processing, and storage can equally influence the accuracy of resulting 3D-printed models. However, the absence of standardized accuracy testing necessitates a cautious interpretation of the findings.

All the examined printers represent newly developed models crafted for applications in dental technology. Digital light processing (DLP) printers, featured in this study, harness light emitted from an LCD screen to initiate the polymerization process of a liquid resin. This intricate procedure entails utilizing a digitally controlled mirror device to direct the light, catalyzing resin polymerization through an oxygen-permeable sheet made of FEP (fluoropolymer), which creates a designated “dead zone”. This zone impedes polymerization, thus allowing for a gap between the foil and the polymerized object surface [41]. Subsequent to printing, DLP polymerization technologies entail specific post-printing procedures, comprising four primary steps: removal from the build platform, cleansing, post-polymerization, support removal, and final refinement [40]. It is imperative to adhere strictly to the post-processing stages as delineated by the manufacturer’s guidelines, tailored to the specific combination of 3D printer and material employed.

The process of removing the dental object from the build platform involves delicately detaching it, typically employing a sharp or planar tool. At this juncture, owing to the nature of the printing technology, the device is encased in uncured resin [40].

Subsequent to removal, the dental object undergoes cleaning to eliminate any residual uncured resin. This cleaning process entails immersion in a solvent bath suitable for the resin, commonly comprising isopropyl alcohol, tripropylene glycol monomethyl ether, or, more recently, water for water-washable resins [42,43,44].

Curing, or post-polymerization, involves subjecting the dental device to UV light with the same wavelength but higher intensity, facilitating complete polymerization. This process enhances the degree of conversion while reducing residual monomer content and reinforcing inner-layer adhesion [45,46].

Upon the completion of curing, the final finishing phase commences, which entails the removal of supports using cutting instruments, disks, or ultrasonic tools, depending on the type of polymer utilized.

Manufacturers typically provide guidelines or standardized protocols detailing optimal printing parameters, support structures, and post-processing techniques tailored to their specific printer or material. However, the flexibility inherent in the technology allows for a certain degree of material compatibility across different wavelengths. Consequently, the expertise of the user plays a pivotal role in crafting a non-standardized protocol [47,48]. It is noteworthy that printing settings and post-processing protocols can significantly influence various aspects [33,34,36,42,47,49], including surface roughness [50], internal fit [51], and physical properties [52] to varying extents. This calibration process necessitates meticulous attention from both the dental office and the collaborating dental laboratory to ensure minimal errors. Each case mandates individualized calibration, contingent upon the magnitude and position of the prosthetic work. This calibration process draws upon the accumulated experience with each specific 3D printing system, allowing for the fine-tuning and optimization of the digital workflow to achieve optimal outcomes. All the specialized articles, studied and described in the Section 4 , constituted a solid starting point for our research. In addition, we carried out a suite of accurate tests in order to study the trueness parameters of the 3D printers, reducing any other variables and accurately following the ISO test standard.

## 5. Conclusions

The findings revealed that the deviation in the dimension of the balls from the reference value was clinically inconsequential for all five printers tested, with the maximum deviation ranging between 1.17% and 2.03% (in the case of the Creo C5 printer). This percentage of deviation remained consistent regardless of the position of the ball on the model. The J5 Dentajet and Form 3B were identified as the most accurate among all evaluated 3D printers, with uniform distortions observed in the three analyzed dimensions (anterior b–c, posterior a–d, and oblique a–c).

The gathered values indicate that there are no constraints for any dental applications of the five analyzed 3D printers. The trueness of printing by the new generations of 3D printers is satisfactory for achieving high-precision mastercasts in dental prosthodontics manufacturing. Differences in values obtained from testing do not influence the manufacturing of printed models for dental prosthetic works in current practice, nor do they restrict the use of printers in various clinical applications.

Further research should focus on investigating the technological workflows of various 3D printers (printer + resin) utilized in dentistry, especially those not previously evaluated. The refinement and calibration of the digital workflow protocol are essential for large dental prosthetics encompassing both sagittal and transverse dimensions.

## Figures and Tables

**Table 1:** The distances between the centers of the balls according to ISO standard 20896-1 [17].

Distances between the Centers of the Balls		
a–b: (40 ± 4) mm	OR	a to b: (35 ± 4) mm
b–c: (35 ± 4) mm		b to c: (20 ± 4) mm
c–d: (40 ± 4) mm		c to d: (35 ± 4) mm
a–d: (55 ± 6) mm		a to d: (35 ± 6) mm
a–c: (60 ± 6) mm		a to c: (44 ± 6) mm
b–d: (60 ± 6) mm		b to d: (44 ± 6) mm

**Table 2:** The nominal values of the parameters of the digital test object in Blender software.

Parameter	Value
a–b	32 mm
b–c	19.474 mm
c–d	32 mm
a–d	38 mm
a–c	42 mm
b–d	42 mm

**Table 3:** Summarizing the specifications of 3D printers and the resins used.

Printer	Anycubic Mono X 6Ks	Asiga Max	Creo C5	Form 3B	J5 DentaJet
Manufacturer	Hongkong Anycubic Technology Co., China	Asiga, Australia	Planmeca Oy, Finland	Formlabs Inc., USA	Stratasys, USA
Technology	mSLA	DLP	DLP	SLA	Polyjet
Wavelength	405 nm	385/405 nm	385 nm	405 nm	NA
XY Resolution	34.4 microns	62 microns	50 microns	25 microns *	18.75/20.625 microns
Layer Height	50 microns	25 microns	50 microns	50 microns	18 microns
Software	Chitubox Basic	Asiga Composer	Planmeca Creo C5 Studio	PreForm	GrabCAD Print
Resin	White-Colored UV Resin Anycubic	DentaMODEL, Asiga	FotoDent model 2, Dreve Dentamid	Model Resin, Formlabs	TrueDent White, Stratasys
IPA Wash	10 min immersion in 99% IPA	10 min immersion in 99% IPA + 10 min immersion in 99% fresh IPA	2 cycles of 6 min each in fresh 99% IPA	10 min immersion in 99% IPA	30 min immersion in 99% IPA **
Curing	10 min in the Form Cure, without heating	Asiga Flash for 20 min, no heating	12 min in the Form Cure, without heating	5 min at 60 °C in the Form Cure	2 h while immersed in glycerol at 80 °C **

* Movement increments of 25 microns, while the laser spot size is 85 microns. ** Only part of the postprocessing protocol, which involves more stages.

**Table 4:** Measurement data results.

ISO Values	Nominal Values	Anycubic Monox 6KS 50 m	Asiga 25 m	Asiga 75 m	Creo c5 Monocrom 50 m	Formlabs 3B 50 m	Stratasys 21
a = 6 ± 2	6	5.922	5.958	5.880	6.110	5.952	5.944
b = 6 ± 2	6	5.986	5.944	5.880	6.070	5.984	5.962
c = 6 ± 2	6	5.974	5.950	5.880	6.122	5.986	5.948
d = 6 ± 2	6	5.904	5.936	5.856	6.102	5.948	5.976
a–b = 35 ± 4	32	31.657	31.933	31.882	31.845	31.982	32.027
b–c = 20 ± 4	19.474	19.306	19.388	19.358	19.367	19.427	19.503
c–d = 35 ± 4	32	31.647	31.931	31.877	31.841	31.964	31.995
a–d = 35 ± 6	38	37.591	37.876	37.845	37.852	37.888	38.086
a–c = 44 ± 6	42	41.566	41.880	41.818	41.772	41.958	42.039
b–d = 44 ± 6	42	41.563	41.882	41.822	41.811	41.907	42.045

**Table 5:** Calculation of trueness and trueness deviation of ISO standard 20896-1 printed parameters.

Parameter	Anycubic Monox 6KS 50 m		Asiga 25 m		Creo c5 Monocrom 50 m		Formlabs 3B 50 m		Stratasys 21	
	Trueness	Trueness Deviation	Trueness	Trueness Deviation	Trueness	Trueness Deviation	Trueness	Trueness Deviation	Trueness	Trueness Deviation
a	98.70%	−1.30%	99.30%	−0.70%	101.83%	1.83%	99.20%	–0.80%	99.07%	–0.93%
b	99.77%	−0.23%	99.07%	−0.93%	101.17%	1.17%	99.73%	−0.27%	99.37%	−0.63%
c	99.57%	−0.43%	99.17%	−0.83%	102.03%	2.03%	99.77%	−0.23%	99.13%	−0.87%
d	98.40%	−1.60%	98.93%	−1.07%	101.70%	1.70%	99.13%	−0.87%	99.60%	−0.40%
a–b	98.93%	−1.07%	99.79%	−0.21%	99.52%	−0.48%	99.94%	−0.06%	100.08%	0.08%
b–c	99.14%	−0.86%	99.56%	−0.44%	99.45%	−0.55%	99.76%	−0.24%	100.15%	0.15%
c–d	98.90%	−1.10%	99.78%	−0.22%	99.50%	−0.50%	99.89%	−0.11%	99.98%	−0.02%
a–d	98.92%	−1.08%	99.67%	−0.33%	99.61%	−0.39%	99.71%	−0.29%	100.23%	0.23%
a–c	98.97%	−1.03%	99.71%	−0.29%	99.46%	−0.54%	99.90%	−0.10%	100.09%	0.09%
b–d	98.96%	−1.04%	99.72%	−0.28%	99.55%	−0.45%	99.78%	−0.22%	100.11%	0.11%

## Data Availability

The original contributions presented in the study are included in the article/Supplementary Material; further inquiries can be directed to the corresponding authors.

## Supplementary Materials

The following supporting information can be downloaded at: https://www.mdpi.com/article/10.3390/jfb15080210/s1 Table S1. Characteristics of the work equipment (Nikon XTH225 ST). Table S2. Characteristics of the work equipment (Nikon XTH225 ST).
