# Supplementary material for: The Evaluation of the Trueness of Dental Mastercasts Obtained through Different 3D Printing Technologies
Source: J Funct Biomater. 2024 Jul 29;15(8):210. doi: 10.3390/jfb15080210 (PMC11355515; doi:10.3390/jfb15080210)
Supplement: Supplementary file 1 [file jfb-15-00210-s001.zip › jfb-3070020-supplementary.pdf]

Supplementary Materials

Table S1. Characteristics of the work equipment (Nikon XTH225 ST).

**Detector**	Model			PerkinElmer XRD 1620		
	Detector size			409.6 × 409.6 mm		
	Resolution			2048 × 2048 pixels		
	Pixel size			200 µm		
**Parameters**	Beam energy	Beam current	Exposure	Filter	Projections	Frames/Projection
	165 kV	165 µA	1 s	0.45 mm Cu	1440	2

Table S2. Characteristics of the work equipment (Nikon XTH225 ST).

	**Used Values**					
**Parameters**	Beam energy	Beam current	Exposure	Filter	Projections	Frames/Projection
	165 kV	165 µA	1 s	0.45 mm Cu	1440	2
